# Nanoparticle delivery systems for HIV pre-exposure prophylaxis (PrEP): advances and challenges

**DOI:** 10.3762/bjnano.17.60

**Published:** 2026-07-07

**Authors:** Sonia Zahara, Björn M Reinhard

**Affiliations:** 1 Dept. of Chemistry and The Photonics Center, Boston University, Boston, MA 02215, United States

**Keywords:** HIV, nanoparticle, PrEP, targeted delivery

## Abstract

HIV pre-exposure prophylaxis (PrEP) encompasses antiviral drugs or formulations that aim to prevent the establishment of a permanent infection if exposure occurs. Conventional oral PrEP approaches require a daily drug regimen for effective prophylaxis, which can become burdensome, and pill fatigue creates challenges for adherence. There is, consequently, interest in the development of long-acting formulations of antiretrovirals that provide protection over extended periods of time after a single treatment. Nanoformulations and nanoparticle delivery strategies play an important role in achieving long acting (LA)-PrEP. This manuscript reviews emerging nanoparticle delivery platforms for LA-PrEP with a particular focus on four distinct classes of nanomaterials, namely, polymeric nanoparticles, lipid-based nanoparticles, inorganic nanoparticles, and hybrid nanoparticles. Nanoparticle design considerations and targeting strategies for the individual classes are examined, and the opportunities and challenges for the different nanomaterials approaches in the context of LA-PrEP are discussed.

## Review

### Introduction

The facts that an estimated 40.8 million people are currently living with HIV and new infections occur at a rate of approx. 1.3 million per year underline that HIV/AIDS is still a major global health crisis [1]. The Joint United Nations Program on HIV/AIDS (UNAIDS) reported that 31.6 million patients used antiretroviral (ARV) therapy (ART) in 2024 [1]. Despite significant progress in the management of HIV, more efforts are necessary to further limit the spread of HIV through prophylaxis. HIV pre-exposure prophylaxis (PrEP) involves the use of ARV medication by HIV-negative individuals at high risk of exposure. It is one important component in the global effort to eliminate HIV. First-line PrEP options approved by the FDA include oral daily tenofovir disoproxil fumarate/emtricitabine (TDF/FTC) or tenofovir alafenamide/emtricitabine (TAF/FTC) [2–3]. Studies have demonstrated that a daily oral administration of these PrEP medications can reduce the risk of HIV transmission by over 90% among patients who take the medication consistently [2,4–6]. These medications have excellent safety profiles but require daily adherence to oral medication, which can be compromised by factors such as pill burden, fatigue, stigma surrounding daily medication use, and limited access to healthcare [7]. Long-term adherence, in particular, is a challenge [7–9]. In a randomized controlled trial involving young African women (HIV Prevention Trials Network (HPTN) 082), Celum et al. evaluated PrEP uptake, persistence, and long-term adherence [9]. They reported that, although persistence was relatively high and more than 50% of the participants maintained refills for 12 months, the actual adherence declined over time. Only ≈21% achieved high adherence during the first six months, and drug detection further declined by month 12.

Considering these challenges, there is significant interest in long-acting (LA) ARV systems as a strategy to mitigate adherence problems in HIV PrEP [7,10–12]. Effective LA-ARV formulations that require administration once every few months under medical supervision can address many issues related to adherence. Nanoparticles play a key role in achieving this goal. Nanosuspensions, for instance, are used to induce depots of poorly soluble ARVs at the injection site that then achieve a continuous release of free drug over an extended period of time. LA cabotegravir (CAB) developed as an intramuscular (IM) nanosuspension by ViiV Healthcare is an example, and it was the first FDA-approved injectable PrEP product (Apretude) for use in adults and adolescents who weigh at least 35 kg [13–15]. HPTN 083 and 084 are phase-3 trial studies for injectable CAB as a LA-PrEP compared to daily oral TDF/FTC for HIV prevention [14,16–18]. These trials were structured into three phases, an initial “oral-tablet lead-in phase”, followed by an “injection phase”, and finally a “tail phase”. In the first lead-in phase, the participants were given oral tablets for five weeks daily. In the injection phase, participants of the CAB received an initial single 3 mL intramuscular (IM) injection of CAB nanosuspension at week 5 of the lead-in phase, a second injection at week 9, and injections every eight weeks thereafter [16–17]. Both trials, HPTN 083 and 084, demonstrated that IM injections of CAB proved more effective than daily oral TDF/FTC and reduced HIV infection risk by 66% (HPTN 083) [14] and 88% (HPTN 084) [17]. However, this approach also has some limitations and complications, including the need for high IM injection volumes (≥3 mL), the inability to remove the dose after injection in case of adverse reactions or change in preference, and a long-term pharmacokinetic tail after discontinuation that lasts up to 43.7 weeks in men and 67.3 weeks in women (HPTN 077) [7,19–20]. More recently, FDA has approved lenacapavir (Yeztugo) as another injectable LA-PrEP, which is currently the only once-in-six month injectable LA-PrEP option [21–23]. Currently FDA-approved PrEP medications are listed in Table 1.

**Table 1:** Summary of currently approved PrEP medications, including drug class, mechanism of action, and route of administration [3,24].

Generic name/ brand name	Drug class	Mechanism of action	Molecular formula/ PubChem CIDs	FDA approval for PrEP	Route of administration

Tenofovir disoproxil fumarate + emtricitabine (TDF+FTC)/ Truvada	NRTI combination	inhibits HIV reverse transcriptase, preventing viral DNA synthesis and replication	C_31_H_44_FN_8_O_17_PS/ 11954236	2012	daily oral pill
Tenofovir alafenamide + emtricitabine (TAF+FTC)/ Descovy^a^	NRTI combination	inhibits HIV reverse transcriptase, preventing viral DNA synthesis and replication	C_29_H_39_FN_9_O_8_PS/ 90469070	2019	daily oral pill
Cabotegravir/ Apretude	INSTI	blocks HIV integrase, preventing integration of viral DNA into the host cells and viral replication	C_19_H_17_F_2_N_3_O_5_/ 54713659	2021	intramuscular injection every 2 months
Lenacapavir/ Yeztugo	capsid inhibitor	disrupts HIV capsid function, interfering with viral replication and resulting in noninfectious capsids	C_39_H_32_ClF_10_N_7_O_5_S_2_/ 133082658	2025	subcutaneous injection every 6 months

^a^Descovy is not currently approved as oral PrEP for individuals at risk via receptive vaginal sex.

**Figure 1:**
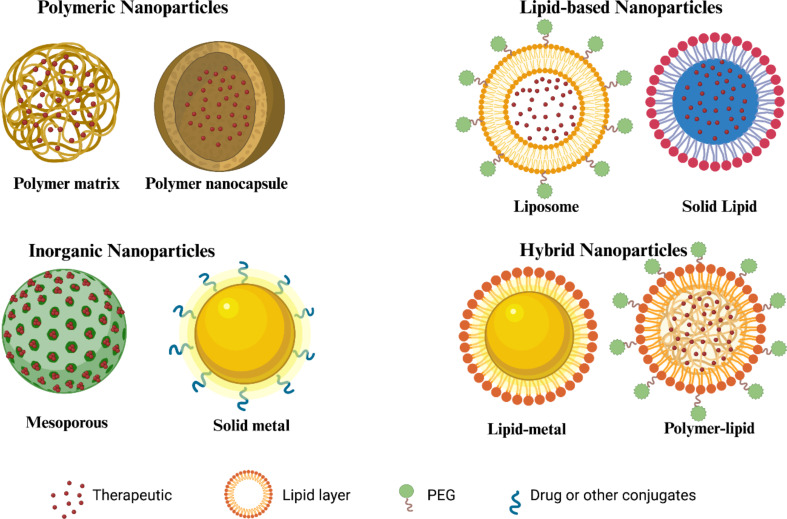
Schematic representation of nanoparticles used in PrEP based on type of material. Created in BioRender. Zahara, S. (2026) https://BioRender.com/g6ld9a6. This content is not subject to CC BY 4.0.

Although injectable ARV nanosuspensions have expanded HIV prevention options beyond daily pills, there is continued interest in the development of additional LA technologies based on nanoparticle delivery systems, especially in combination with active or passive targeting strategies. Nanoparticles are promising delivery systems for concentrating ARVs in tissues that are entry sites for HIV during sexual intercourse and secondary lymphoid tissues, which play an important role in the spread and establishment of the infection. In addition to providing opportunities for tissue-specific targeting, nanoparticle-based delivery systems are also compatible with LA-PrEP. They can be designed to encapsulate both hydrophobic or hydrophilic drugs, protect drugs from enzymatic degradation, and control release kinetics. Nanoparticle platforms that combine the capacity to hold a high payload of ARVs with the ability to carry it to specific sites and release it locally, are of interest for PrEP. Moreover, nanoparticles can be engineered to traverse biological barriers and can be administered via multiple routes, including IM, transdermal, subcutaneous, and mucosal pathways [25–27]. Each administration route has its particular requirements in terms of size, mechanical properties, and surface composition that need to be considered in the design of the nanoparticles. The choice of core material plays a pivotal role in defining the physicochemical properties, release behavior, biocompatibility, and clinical feasibility of nanoparticle formulations. Various material classes have been explored for HIV PrEP applications, each offering unique advantages and limitations depending on the route of administration, duration of action, and drug type. In this review, we focus on nanoparticle delivery platforms for PrEP. To better understand the landscape of nanoparticle-based HIV prophylaxis delivery strategies, we have broadly categorized existing platforms into four main categories based on their core material (Figure 1). (1) Polymeric nanoparticles: These nanoparticles are designed from biodegradable polymers such as poly(lactic-*co*-glycolic acid) (PLGA), polycaprolactone (PCL), or cellulose acetate phthalate (CAP). These systems are known for their controlled degradation and tunable drug release profiles. Their ability to sustain therapeutic levels over extended periods of time makes them particularly suitable for both systemic and topical LA-PrEP. (2) Lipid-based nanoparticles (LBNPs): These include liposomes, lipid nanoparticles (LNPs), solid lipid nanoparticles (SLNs), and nanostructured lipid carriers (NLCs). LNPs have a less ordered internal structure than liposomes and are primarily used for nucleic acid delivery [28]. In general, LBNPs can encapsulate a wide range of drug types, and some of these nanoparticles share similarities with biological membranes. Their excellent biocompatibility and mucosal tissue affinity position them as strong candidates for mucosal PrEP applications. (3) Inorganic nanoparticles: This group covers nanoparticles from materials such as gold or mesoporous silica and silicon. Inorganic platforms offer distinct properties like structural rigidity, potential use as adjuvant systems, and some studies reported virion removal by attachment [29]. Although less widely applied in PrEP, they are attracting interest in microbicidal and vaccine-focused strategies. (4) Hybrid nanoparticles: These systems are composed of multiple materials, for example lipid–polymer hybrids and magnetoliposomes. The combination of two or more materials provides opportunities for enhancing PrEP relevant nanoparticle properties, including improved drug loading and controlled release.

**Figure 2:**
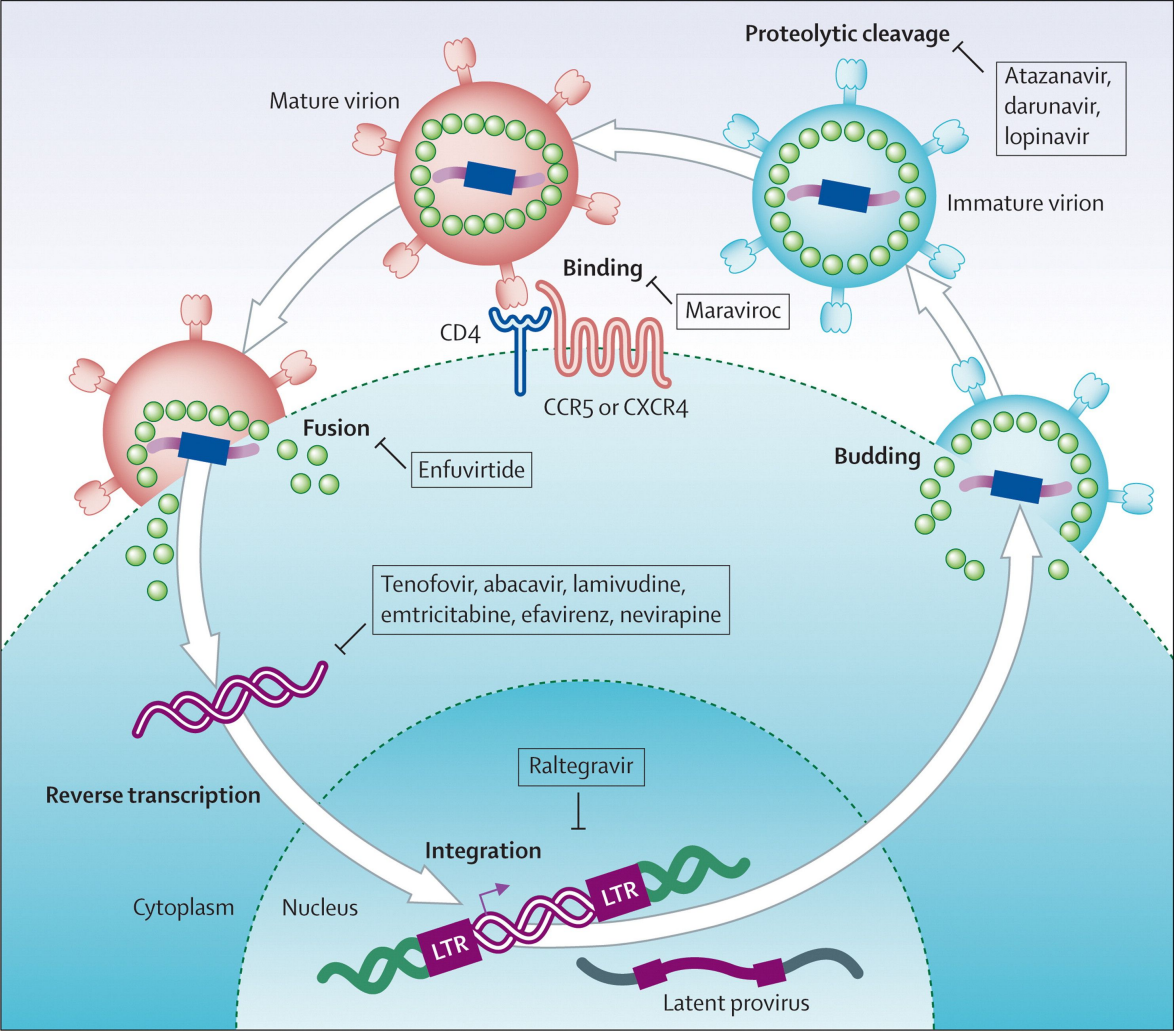
Overview of the HIV life cycle and key stages targeted by antiretroviral drugs. Figure 2 was reprinted from [38], the Lancet, vol. 376, by P. A. Volberding; S. G. Deeks, “Antiretroviral Therapy and Management of HIV Infection”, pages 49–62, Copyright (2010), with permission from Elsevier. This content is not subject to CC BY 4.0.

PrEP nanoparticle delivery platforms can be equipped with different functionalization and passivation chemistries. The surface properties determine colloidal stability as well as the interactions between the nanoparticles and the surrounding. These properties are critical for the nanoparticles’ ability to target specific tissues and cells. CD4^+^ T cells and antigen-presenting cells of myeloid lineage, such as macrophages or dendritic cells (DCs), are involved in HIV infection and are, therefore, cellular targets of interest. CD4^+^ T cells are the primary host cells of HIV in cis-infection, in which the virus directly binds to a host cell and initiates replication [30], whereas myeloid cells play a role in trans-infection [31–33]. In trans-infection, the virus binds to myeloid cells via a myeloid-cell receptor, in particular sialic acid-binding Ig-like lectin 1 (Siglec-1/CD169), which then transfer the virus to its actual CD4^+^ host cell in secondary lymphoid tissues [34–36]. Once the virus has bound to a host cell via the CD4 receptor, it goes through different stages of its infection cycle, including fusion, reverse transcription, integration, translation, budding, and maturation of newly formed viruses (Figure 2) [37–38]. The ARV drugs used for PrEP work by blocking a critical step in the HIV life cycle, such as reverse transcription or integration, thereby stopping an infection before it can take hold. Current ARVs used for this purpose often include a combination of TDF/FTC as nucleoside reverse transcriptase inhibitors (NRTIs) or long-acting injectable CAB as integrase strand transfer inhibitor (INSTI) [2,15–16].

Targeting cells relevant for both cis- and trans-infection through appropriate nanoparticle delivery strategies is of interest for enhancing the prophylactic effect of ARV drugs. In the following, we will subdivide our discussion of targeted and non-targeted delivery approaches for each nanoparticle category 1–4 and highlight representative cases for both strategies.

### Polymeric nanoparticles

Polymeric nanoparticles have emerged as a versatile platform for LA ARV delivery due to their tunable biodegradability and ability to encapsulate a wide range of drugs. Polymeric nanoparticle systems can be designed as injectables, microneedle patches, implants, or topical films and gels, allowing them flexibility in administration routes beyond IM requirements of nanosuspensions [39–44]. Moreover, the increased biocompatibility and controlled degradation ability of polymers, such as PLGA, reduce the long-term toxicity concerns that are associated with some inorganic particles that exhibit no or slow biodegradation such as gold or silica [45–47]. Furthermore, polymers can be engineered to respond to environmental triggers such as pH or temperature for smart drug release [39–40,42]. This provides polymeric systems additional functionalities beyond targeted delivery to HIV-susceptible tissues or cell populations [41–44].

A major advantage of polymeric platforms lies in their ability to take up and store drug cargo in their interior with a capacity that depends on the chemical nature of the polymer and the structure of the nanoparticle. They provide engineering degrees of freedom to tune release properties and thus generate opportunities for developing systems that can demonstrate sustained drug release over extended periods of time [48]. Unlike dense solid nanoparticles such as gold, polymeric nanoparticles have an internal volume that can accommodate substantial loading with drug cargo, typically dispersed throughout the polymer matrix. This property is particularly beneficial for drugs with poor bioavailability or short plasma half-lives, as it allows a reduction in dosing frequency and improves adherence. In some designs, drug cargo is confined within nanocapsule-like structures formed by a polymer shell [49]. The core of these nanocapsules can be hollow or consist of a distinct liquid or solid material [49–51], thus making it able to carry different drugs, although the applicability of this approach to PrEP requires further validation.

Several studies have demonstrated the effectiveness of polymeric nanoparticles in delivering ARVs with improved pharmacokinetics and HIV prevention efficacy. For instance, Destache et al. designed TDF-loaded PLGA nanoparticles in a thermosensitive gel (TDF-NPs-TMS gel) to improve vaginal gel-based HIV PrEP systems [42]. When the TDF-NPs-TMS gel with 0.1% or 0.5% TDF was applied intravaginally to humanized mouse models 4 or 24 h before viral challenge, respectively, it fully protected the mice for at least 4 weeks. However, mice that were treated with TDF-NPs-TMS gel with 1% TDF and challenged after 1 week became HIV positive two weeks after inoculation. The protection of mice even when challenged 24 h after gel application demonstrates that the PLGA-based system has the potential for localized PrEP for at least 24 h after dosing. In another study, also focused on localized mucosal drug delivery, the INSTI dolutegravir (DTG) was encapsulated into nanoparticles made from CAP, which is a pH-sensitive polymer with intrinsic HIV-1 entry inhibitory activity [39]. The resulting pH-responsive CAP-DTG nanoparticles were incorporated into a thermosensitive gel for vaginal application. This resulted in a pH-responsive release of drug, demonstrating limited release of DTG at acidic pH (4.2), representative of vaginal environment, and significantly enhanced drug release at neutral pH (7.4), simulating semen exposure. The in vitro studies also showed cellular uptake of CAP nanoparticles into vaginal epithelial cells and sustained intracellular presence over 7 days. The CAP-DTG nanoparticle containing gel could be a promising strategy for LA topical PrEP due to its ability to provide pH-responsive release of ARV drugs localized to the potential exposure site while minimizing systemic absorption of nanoparticles and drugs.

In another recent approach to polymeric drug delivery, Zhang et al. explored the use of dissolving polymeric microneedles loaded with nanoparticles of the potent ARVs bictegravir (BIC) and TAF [41]. BIC blocks HIV transmission by targeting HIV integrase while TAF targets HIV reverse transcriptase. These microneedles were designed for transdermal delivery of ARVs that offers a minimally invasive alternative to traditional injections. This study demonstrated that in Sprauge–Dawley rats, microneedle application of BIC achieves a pharmacokinetics profile comparable to IM injection. Moreover, the maximum concentration (*C*_max_) or area under curve (AUC) showed no significant difference compared to IM injection. Although the overall exposure was lower than for IM injection, the plasma levels of BIC were sustained above the inhibitory threshold for up to 21 days showing strong potential for its use in LA-PrEP. The study investigated TAF, and further evaluation of the intracellular active metabolite tenofovir (TFV) diphosphate is required to determine the true potential for LA-PrEP applications. Nevertheless, the study by Zhang et al. illustrates the potential of polymer-based, dissolving microneedles for LA-PrEP and provides opportunities for overcoming longstanding challenges associated with traditional depot formulations, such as lack of control of dosing after injection, side-effects, and overall invasiveness.

Mandal et al. demonstrated that the encapsulation of BIC+TAF in PLGA nanoparticles prolongs drug retention and decreases cytotoxicity of free TAF and BIC [43]. The authors observed that the BIC+TAF nanoparticles achieved a drug selectivity index that was 472 times higher than for the free drugs solution when tested with human peripheral blood mononuclear cells (PBMCs). Moreover, when Balb/C mice were subcutaneously injected with BIC+TAF nanoparticles, both drugs showed prolonged persistence in key HIV target tissues including spleen, vagina, colon, and lymph nodes (LNs). In addition, BIC and TFV (TAF metabolite) concentrations were maintained above the half maximal inhibitory concentration (IC_50_) for at least 30 days in plasma as well as in LNs and spleen. Another study using polymeric nanoparticles demonstrated that nanoencapsulation helps to lower IC_50_ values and sustain prolonged drug levels, which may help to mitigate the pharmacokinetic challenges of conventional ARV systems [48]. FTC is one of the first-line approved PrEP by FDA, but it requires frequent dosing to maintain efficacy due to its short plasma half-life (8–10 h) and a large volume of distribution (≈1.4 L/kg) [48,52]. To address these limitations, Mandal et al. developed FTC-loaded PLGA nanoparticles that achieved significantly higher drug retention in TZM-bl cells after 48 and 96 h treatment intervals compared to free drug solution [48]. When the drug release was tested under endosomal pH conditions (pH 5.5), the FTC-NPs achieved a sustainable release for up to 30 days after an initial burst release within the first hour. Notably, the nanoformulated FTC exhibited a lower IC_50_ compared to the free drug, indicating enhanced antiviral potency, presumably due to enhanced intracellular drug delivery and retention. This study highlights that the encapsulation of FTC or other drugs with similar chemical properties may improve sustained drug release and provide a potential LA-PrEP strategy for less frequent dosing without increasing cytotoxicity.

Despite the outlined advantages, polymeric systems also face some challenges and limitations. For one, nanoparticles can aggregate, and this negatively affects delivery applications. Furthermore, the use of organic solvents during their fabrication may raise some concerns about residual solvent toxicity [53]. Another point to note is that active targeting via surface ligand decoration, common in lipid-based systems, is more challenging to implement with polymers and typically requires additional chemical modification of the polymer building blocks. The degradation kinetics of polymer nanoparticles depends on various conditions, including the pH or the concentration of degrading enzymes. As these factors can differ between patients, they can create some variability in the pharmacokinetics between individuals [47,54]. Despite remaining challenges, polymeric nanoparticles with their widely tunable properties are a promising class of materials to facilitate LA-PrEP and selective targeting of ARVs to relevant tissues. Mannosylated PLGA nanoparticles even showed some promise to improve drug uptake in the brain macrophages compared to free drug solutions [55]. This effect may be attributed to mannose receptor-mediated uptake into macrophages, but the exact mechanism of blood–brain barrier (BBB) transfer has not been determined.

### Lipid-based nanoparticles

LBNPs, including liposomes, LNPs, SLNs, and NLCs, represent a well-established class of nanocarriers with clinical applications in drug delivery and have also been explored for HIV prophylaxis. These lipid-based systems are typically composed of natural or synthetic lipids and surfactants that self-assemble into vesicular or lipid–core structures. Because of their modular design, LBNPs can encapsulate both hydrophilic and hydrophobic ARVs. Moreover, the ease of surface functionalization with targeting ligands such as glycosphingolipids, peptides, or antibodies makes them an interesting option for targeted delivery to HIV-relevant immune cells, including CD4^+^ T cells. Immune cells not only serve as initial targets during transmission but also contribute to the establishment of viral reservoirs. Viral reservoirs are specific cells, such as resting memory CD4^+^ T cells and tissue macrophages, where HIV can remain latent, hidden from both the immune system and ART [56–57]. Compared to polymeric systems, LBNPs in general allow for a higher flexibility in tuning surface charge and membrane composition to facilitate interactions with immune cells [56]. Both factors enhance the overall ability of targeted delivery.

One example of surface functionalized LBNPs for targeted delivery are DRV-loaded SLNs surface-conjugated with a peptide with affinity for CD4 receptors [58]. The clinical use of DRV has been limited by its low bioavailability largely due to its lipophilic nature. First pass metabolism and efflux transport further reduce bioavailability [59]. To address these limitations, DRV-loaded SLNs were designed to improve delivery to HIV reservoirs [58]. In permeability studies, the nanoparticles showed a fourfold increase in transport across a Caco-2 cell membrane compared to plain drug solution. Furthermore, the peptide-grafted SLNs achieved enhanced cellular uptake in a CD4^+^ T cell model (MOLT-4) compared to cell models without CD4 receptor. A comparison of the relative bioavailability of DRV achieved by DRV-loaded SLNs versus free drug suspension after oral administration to male Wister rats demonstrated enhanced (569%) bioavailability of DRV for DRV-SLNs in vivo. Moreover, DRV-SLNs achieved higher concentrations of the drug in potential HIV reservoir organs, such as spleen and brain. Expanding the ligand functionalization strategy, another study developed a CD4-targeted LNP system designed for the delivery of mRNA rather than ARVs in which the nanoparticles were functionalized with anti-CD4 antibodies [60]. When the particles were intravenously administered to mice, CD4-targeted mRNA-LNPs bound to T cells in spleen and produced approx. 30-fold higher reporter mRNA signals in these cells than mRNA-LNPs controls that lacked targeting. While this system was not specifically developed for PrEP, it still illustrates the broader potential of ligand-functionalized LBNP platforms for use in CD4-targeting nanomedicine and future HIV prevention strategies. Continuing on the development of receptor-targeting LBNPs, Patel et al. developed rilpivirine (RPV)-loaded LBNPs functionalized with the CCR5 binding peptide D-Ala-Ser-Thr-Thr-Thr-Asn-Tyr-Thr-NH_2_ (LBNP-RPV-CCR5) [61]. CCR5 is a co-receptor for HIV entry into host cells that is expressed on the surface of various immune cells, including CD4^+^ T cells and macrophages. When nanoparticle uptake was tested in monocyte derived macrophages (MDMs) in vitro, LBNP-RPV-CCR5 showed enhanced cellular uptake compared to LBNPs without the ligand, resulting in higher intracellular RPV retention that enabled prolonged suppression of HIV replication. Biodistribution studies demonstrated enhanced accumulation of LBNP-RPV-CCR5 in spleen after tail vein injection, which may be attributed to the presence of a high number of CCR5-presenting immune cells in spleen [61]. Moreover, the authors also demonstrated increased accumulation of LBNP-RPV-CCR5 in human microglia and higher retention of RPV in brain tissue when humanized mice were intravenously injected under facilitated conditions to induce BBB disruption. Importantly, in HIV-1ADA-infected humanized mice, LBNPs-RPV-CCR5 controlled viral growth better than LBNP-RPV and showed improved viral suppression with reduced plasma viral RNA levels and decreased HIV-1 p24 antigen expression across multiple organs. These studies highlight that ligand functionalization of nanoformulations not only improves the drug accumulation in relevant tissues but also helps maintaining the therapeutic levels over extended time duration.

Targeted long-acting combination ART (TLC-ART) is another promising strategy for HIV treatment and prevention where a combination of ARVs is co-encapsulated in nanoformulations to achieve sustained intracellular and plasma levels, with preferential delivery to HIV target tissues and cells. In the so-called “TLC-ART 101”, TFV, lopinavir (LPV), and ritonavir (RTV) were co-formulated in a lipid-stabilized nanosuspension and administered subcutaneously to macaques [62]. TFV and LPV showed persistent drug levels in plasma and PBMCs for over two weeks. Lymph node mononuclear cells had up to ≈79-fold higher intracellular drug levels than PBMCs, depending on drug type and time point. In a subsequent study of TLC-ART 101, Ho and coworkers replaced LPV with atazanavir (ATV) but kept TFV and RTV [63]. The TLC-ART nanoparticle system with ATV showed LA behavior with preferential lymphoid tissue accumulation for all three drugs. While the pharmacokinetics of ATV showed some differences to that of LPV, all three compounds achieved successful enrichment in lymphocytes, and RTV and TFV showed similar pharmacokinetics to those observed with TLC-ART 101. These observations suggest that this nanoparticle platform has potential for accommodating various ARV combinations [63].

Beyond systemic and cellular targeting, LBNPs have also been engineered for topical applications via hydrogel matrices. In a study by Faria et al., a liposomal hydrogel platform was developed for vaginal delivery of co-loaded ARV drugs aiming to maintain effective drug levels directly at the site of viral entry that can be particularly useful for the pericoital protection of females [64]. In this system TDF encapsulated in liposomes and FTC were incorporated into a hydrogel matrix. In vitro studies showed that this formulation provides rapid initial release of FTC (40% of total FTC within the first hour), ensuring rapid availability of FTC after vaginal administration followed by a sustained release of both drugs, but especially of TDF, for up to several hours [64]. Although this platform does not involve active targeting or systemic delivery, it still highlights the versatility of lipid-based nanocarriers for topical PrEP applications. In another recent study, a SLN gel formulation of LPV was developed to overcome the poor oral bioavailability of LPV and thus to improve its systemic bioavailability [65]. The LPV-SNL-based gel showed improved performance compared to both the plain gel and oral suspension. Ex vivo skin permeation of the LPV-SNL gel demonstrated sustained release with ≈71% drug release over 12 h compared to nearly complete release (≈98%) at the end of 10 h for the plain LPV gel. *C*_max_ and AUC were also higher for the LPV-SLN gel when topically applied in rats compared to plain LPV gel and oral LPV. This indicates improved bioavailability. While LPV is not used as a PrEP drug, this study still demonstrates that SLN gels may offer an alternative route towards improved drug penetration and systemic distribution of PrEP compounds with similar oral limitations.

In addition to the classical anti-viral applications, recent innovations have expanded LBNP use toward mRNA-based HIV prophylaxis. One study used LNPs to deliver mRNA that encoded a stabilized, cleavage-independent, native-like HIV-1 envelope (Env) trimer [66]. These nanoparticles were designed to induce the expression of HIV-1 Env trimers on host cell surfaces so that the immune system could recognize the protein in its native-like form and generate neutralizing antibodies. In rabbits, this approach generated an autologous tier-2 neutralizing antibody response at levels comparable to those achieved using adjuvanted, sequence-matched soluble Env. Although not a conventional ARV-based PrEP system, this system still highlights a potential of LBNP platforms to enable complementary mRNA-based HIV prophylactic strategies.

While LBNPs have demonstrated great utility as PrEP delivery platform, certain limitations remain. The efficacy of LBNPs can be limited by relatively low drug loading capacity and physical instability related to fusion, aggregation, or leakage during storage. Burst release and low encapsulation of certain ARVs, particularly hydrophilic drugs, can also compromise sustained efficacy [67]. Despite these issues, ease of functionalization, innate biocompatibility, and potential for targeted delivery to immune cells make LBNPs an interesting class of nanocarriers for the development of LA-PrEP strategies, especially if continuous sustained release can be accomplished with polymeric materials in hybrid systems.

### Inorganic nanoparticles

Inorganic nanoparticles, particularly those made of metals (e.g., gold, silver) and mesoporous silicon have gained attention as antivirals as well as HIV drug delivery platforms due to their unique physicochemical properties that differ from biodegradable polymers or lipid-based systems. Some metallic nanoparticles (MNPs), such as gold nanoparticles (AuNPs), exhibit long-term stability, chemically modifiable surfaces, and potential for intrinsic antiviral activity [68–69]. AuNPs can, for instance, interfere with and block HIV gp120-CD4 interactions. Other MNPs, like silver nanoparticles (AgNPs) may also show antiviral effects such as inhibiting viral entry and replication [68,70]. The intrinsically high surface-to-volume ratios of these nanoparticles and the capacity for surface functionalization provide the prerequisites for engineering multivalent interactions with targeted biomolecules or cell receptors for optimized active targeting approaches [71].

Kulkarni and co-workers explored a multifunctional AuNP-based system [72]. They tethered the established ARV TFV onto AuNPs via covalent conjugation. The resulting AuNP-TFV conjugates exhibited significantly enhanced anti-HIV-1 activity in TZM-bl and PBMCs. They also tested AuNP-TFV for anti-HIV-1 reverse transcriptase (RT-as) and anti-HIV-1 protease activity. Both enzymes are crucial for HIV-1 replication. AuNP-TFV demonstrated ≈15-fold higher anti-RT-as activity compared to TFV and showed potent anti-HIV-1 protease activity. AuNP-TFV also achieved improved biodistribution in vivo and accumulated in tissues that provide potential HIV reservoirs. These findings corroborate that AuNPs have some potential both as delivery vehicles and potentially also as active therapeutic agents in the context of HIV treatment. However, despite these advantages, the clinical translation of MNPs like AuNPs still requires further research to overcome several key obstacles including risks associated with MNP accumulation, incomplete understanding of long-term clearance, and insufficient mechanistic understanding of the nanoparticles’ intrinsic antiviral actions such as protease inhibition [72–74]. Some studies of the toxicity of MNPs have provided indications of mitochondrial dysfunction, increased membrane permeability, cellular damage, and oxidative stress in host cells in response to MNP exposure [75–76].

Silicon-based systems, particularly mesoporous silicon and silica nanoparticles may overcome some of these drawbacks. They have attracted interest as delivery systems due to their relatively high surface area and drug loading capacity, as well as increased biocompatibility and biodegradability. The nanoparticles gradually dissolve into silicic acid under physiological conditions [77–83]. Silicic acid is generally considered non-toxic, although high local concentrations of acids may still have detrimental effects. In a study by Osminkina et al., mesoporous silicon nanoparticles demonstrated antiviral activity against multiple virus types, including HIV, in vitro through physical adsorption of virions [29]. However, the antiviral effect has been demonstrated primarily in vitro, and potential toxicity and in vivo safety require more evaluation. Furthermore, the extracellular mechanism does not address intracellular viral reservoirs, and the long-term in vivo biodistribution and clearance of silicon nanoparticles, as well as the underlying mechanisms, still need to be investigated further.

Another promising direction in inorganic nanoparticles research is their application in vaccine immunogen delivery. Peterhoff et al. presented a stabilized HIV Env trimer on the surface of nonporous silica nanoparticles (100–200 nm) [84]. They modified the Env for site-specific covalent attachment to the nanoparticles while preserving the native structure and antigenicity, and achieved a multivalent display of Env on the nanoparticles surface. The multivalent presentation of antigenically intact Env resulted in enhanced uptake by dendritic cells and efficient activation of B cells even at low nanomolar concentrations in vitro. Intriguingly, the Env-functionalized nanoparticles induced serum reactivity comparable to that of adjuvanted soluble Env, and in some conditions even without adjuvant. This suggests that the multivalent silica nanoparticles provide a dose sparing effect and may reduce dependence on external adjuvants highlighting the potential of silica nanoparticles to improve vaccine efficacy. The study was, however, limited to short-term immunization studies in mice. Furthermore, the long-term safety and immunogenicity of silica nanoparticles require further investigation.

Despite impressive development, inorganic nanoparticles face some inherent limitations for LA HIV PrEP. Compared to polymeric systems, solid MNPs in particular have smaller payload capacities and often require surface tethering strategies. This makes sustained drug release at least for a subset of inorganic materials challenging. There are also some concerns about long-term accumulation and clearance of some inorganic nanoparticles because of the non-biodegradable nature, especially if administered systemically. Moreover, it is necessary to carefully optimize the formulation parameters due to potential cytotoxicity concerns, especially at higher doses. While mesoporous silicon- and silica-based particles offer improved biocompatibility and degradability, they too require careful optimization to balance efficacy and safety. Overall, inorganic nanoparticles occupy a distinct niche in HIV PrEP research, offering multifunctionality and precision but requiring cautious evaluation for translational use.

### Hybrid nanoparticles

Hybrid nanoparticles are composed of two or more distinct materials whose combinations provide opportunities for enhancing materials properties relevant for LA-PrEP, such as improved stability, enhanced drug loading, controlled release, or targeted delivery to certain immune cell populations or tissue reservoirs. These nanoparticles have emerged as a versatile approach for controlling and treating HIV. The hybrid approach may combine polymers, lipids, or inorganic materials, such as mesoporous silica, into a single platform. Among the possible formulations of hybrid nanoparticles, polymer–lipid hybrid nanoparticles (PLNs) have emerged as a particularly promising class of hybrid systems. These nanocarrier systems are composed of a non-toxic biodegradable polymeric core coated by a lipid layer or lipid membrane [85–89]. An outer PEG layer can be added by covalent attachment to the lipid shell to further improve colloidal stability and prolong systemic circulation by reducing opsonization and clearance. The polymer core can improve drug encapsulation and aids sustained release, while the lipid coating increases biocompatibility, in some cases reduces burst release, and provides new opportunities for surface functionalization with targeting ligands. A straightforward targeting strategy is achieved by incorporating lipids that bind to specific cell surface receptors. For instance, the ganglioside GM3 allows targeting of CD169 [90–93]. This receptor is enriched on myeloid cells that play a role in HIV trans-infection and as potential reservoir sites [33,91,94–95]. The molecular targeting approach allows enrichment of PLNs on specific immune cell subsets implicated in HIV pathogenesis and latency.

We validated PLNs as virus-mimicking nanoparticles that target CD169^+^ macrophages [90,96]. We used biodegradable PLA as core material, encapsulated two ARVs, RPV and CAB, and incorporated GM3 as ligand into the lipid monolayer. The virus mimicking PLNs bound specifically to CD169^+^ macrophages and demonstrated HIV-like trafficking behaviors in vitro. Specifically, the particles avoided endolysosomal degradation pathways and were instead sequestered in so-called virus-containing compartments (VCCs). VCCs are intracellular reservoirs where HIV can persist and evade immune clearance and drug treatment [94,97–98]. GM3-presenting nanoparticles with a PLA core achieved sustained inhibition of HIV-1 replication in primary human macrophages for up to 35 days, demonstrating the potential of this hybrid design for depot-like, targeted LA delivery [90]. Subsequent work with GM3-PLNs showed specific targeting and persistence in lymph node-resident CD169^+^ macrophages in mice [99]. Elkateb et al. also developed various PLN hybrid systems, in which the authors co-loaded with DRV and ritonavir (RTV) [100]. Certain formulations showed enhanced colloidal stability and improved transcellular permeability in vitro.

Similar to our GM3-PLNs work [90], we have also investigated lipid–AuNP hybrids that incorporate GM3 and/or phosphatidylserine (PS) in the lipid membrane [101]. This design allowed the nanoparticles to mimic key aspects of the HIV viral envelope, act as artificial virus nanoparticles (AVNs), and target VCCs within macrophages and dendritic cells [101–102]. The GM3/PS lipid ratio on the nanoparticles surface provided some control over the intracellular trafficking pathways, achieving preferential enrichment of the nanoparticles in either endolysosomal compartments or VCCs [101]. Combined, these studies highlight the importance of lipid composition and ligand decoration on the nanoparticle surface in governing nanoparticle uptake and intracellular fate. GM3-functionalized nanoparticles offer a potential approach for targeted delivery of ARVs directly to latent HIV reservoirs. The payload capacity of GM3-presenting solid MNP is limited, but Zang et al. extended this approach to other inorganic nanoparticles and generated GM3-presenting lipid-coated mesoporous silica nanoparticles co-loaded with CAB and RPV [103].

Hybrid systems have also been explored for topical delivery of ARVs in the context of PrEP. In the case of TFV, studies have shown that insufficient drug penetration of the lower female genital tract can reduce the effect of oral PrEP and decrease the protection [104]. To overcome this, Nunes et al. developed electrospun fibers for topical PrEP, combining either PCL directly loaded with TDF/FTC or a hybrid liposome-in-PVA system in which the drug-loaded liposomes were incorporated into PVA fibers [105]. The authors performed an in vivo pharmacokinetics study in mice in which a single intravaginal administration of the fiber systems was compared with five daily oral doses of TDF/FTC. This study demonstrated generally higher drug concentrations in vaginal lavage for the liposome-in-PVA fibers compared to oral PrEP. PCL fibers also showed superior behavior over oral treatment, but the advantage was smaller. These findings suggest that lipid–polymer hybrid fibers systems can support rapid local drug delivery and may complement daily oral PrEP for HIV prevention.

In a complementary line of research, hybrid nanocarriers have been developed not just for prophylaxis but for HIV reservoir reactivation or latency reversal. Latency reversal refers to the awakening of dormant HIV to force viral expression within cellular reservoirs, such as resting CD4^+^ T cells, so that the infected cells can be recognized by the immune system and/or made susceptible to ART [106]. In one example Cao et al. integrated a combination of multiple mechanistically distinct latency-reversing agents into lipid-coated PLGA nanoparticles that were also designed to target CD4^+^ T cells in lymph nodes [107]. These nanoparticles demonstrated synergistic HIV latency reversal in cell models as well as in CD4^+^ T cells from virologically suppressed patients. The nanoparticles were found to preferentially traffic to lymph nodes after subcutaneous administration and selectively induce activation of CD4^+^ T cells, which was sustained for up to seven days in a mouse model, with stronger effects at earlier time points.

Most HIV nanoparticle studies have so far focused on targeting immune cells in lymphoid tissues, and significant progress has been achieved in this regard. Targeting HIV reservoirs in the brain remains challenging due to the restrictive nature of the BBB. To address this significant challenge, Tomitaka et al. developed magneto-plasmonic liposomes (MPLs), a hybrid nanoparticle system combining magneto-plasmonic nanoparticles and liposomes loaded with TDF for a triple-modality image-guided drug delivery [108]. These MPLs demonstrated improved transmigration across an in vitro BBB model under magnetic guidance, and superior antiviral activity compared to free drug. The reported findings confirm a significant potential for reaching and treating HIV reservoirs in the brain.

Overall, hybrid materials provide a promising strategy for combining the advantages of diverse materials to overcome challenges associated with achieving LA-PrEP through targeted ARV-loaded nanoparticles and to target potential reservoir sites. The higher complexity of the materials is for many applications offset by a gain in versatility that benefits the optimization of targeting and delivery properties.

In closing, we summarize the versatility of nanoparticle applications for PrEP by highlighting selected examples from the four reviewed nanoparticle categories in Table 2. Nanoparticle-enhanced administration and delivery strategies are complemented by immunomodulatory approaches that trigger immune activation and antibody generation. The highlighted examples, together with all other studies reviewed in this manuscript, explore a broad range of unique approaches for improving the efficacy and applicability of long-acting PrEP with potential for limiting the spread of HIV.

**Table 2:** Representative nanocarrier-based HIV prevention and therapeutic strategies classified according to translational relevance.

Type of nanocarrier	Drug(s)/biologics	Model system	Route of administration	Key outcome	Ref.

polymeric	BIC + TAF	Sprauge–Dawley rats	transdermal	sustained BIC levels above inhibitory threshold for up to 21 days using minimally invasive dissolving microneedles	[41]^a^
polymeric	TDF	humanized bone marrow-liver-thymus (hu-BLT) mice	topical (intravaginal administration)	complete protection against HIV-1 challenge after pre-exposure intravaginal administration	[42]^b^
polymeric	BIC + TAF	Balb/c mice	subcutaneous	sustained drug levels above inhibitory threshold for up to 30 days	[43]^a^
lipid-based	RPV	HIV-1ADA-infected humanized (hu) mice	intravenous	enhanced tissue distribution and sustained viral suppression in HIV-1-infected hu-mice, reducing p24 antigen in multiple tissues	[61]^a^
lipid-based	TDF + FTC	in vitro synthetic permeation model	proposed vaginal topical delivery	enhanced TDF permeation with rapid FTC and sustained TDF release for topical PrEP applications	[64]^a^
lipid-based	LPV	porcine ear skin model (ex vivo) and Wistar rats	topical	improved skin permeation and enhanced systemic bioavailability compared to oral suspension and plain gel	[65]^a^
inorganic	TFV	TZM-bl and PBMCs (in vitro) and Balb/c mice	intravenous	enhanced anti-HIV activity, improved cellular uptake, and systemic biodistribution, including organs associated with viral reservoirs	[72]^a^
inorganic	stabilized HIV-1 Env	DCs and VRC01 B cells (in vitro) and C57BL/6J mice	subcutaneous	enhanced dendritic cell uptake and B cell activation, and improved in vivo immunogenicity in mouse model	[84]^c^
hybrid polymeric–lipid	RPV + CAB	macrophages and Balb/c mice	subcutaneous	targeted delivery to CD169^+^ lymph node macrophages in Balb/c mice, prolonged intracellular retention of nanoparticles, and sustained inhibition of cis- and trans HIV infection in vitro	[90,99]^a^
hybrid polymeric–lipid	TDF + FTC	female ICR mouse	topical (intravaginal administration)	single intravaginal application of the fiber systems produced higher vaginal drug concentrations than five daily oral doses of TDF/FTC	[105]^a^
hybrid lipid–inorganic	TDF	HIV-infected CHME-5 cells (in vitro)	in vitro treatment	improved BBB transmigration and therapeutic activity against HIV-infected microglial cells	[108]^a^

^a^Denotes studies demonstrating antiretroviral delivery systems with advantageous pharmacokinetic or biodistribution outcomes; ^b^indicates studies demonstrating in vivo prophylactic efficacy against HIV infection; ^c^denotes vaccine-based approaches involving immune activation and antibody generation.

## Conclusion

Eradicating HIV remains a major global challenge. While current oral ART has proven effective in prevention and treatment, they are often limited by poor adherence, which leads to reduced treatment effectiveness. LA nanosuspensions have significantly improved adherence and prevention outcomes in PrEP, but current strategies are currently limited to a few selected compounds, lack targeting functionalities, and require high doses. Nanoparticle delivery platforms provide opportunities for tissue and even cell-specific targeting as well as improved tissue penetration across biological barriers and are therefore of interest to implement LA-PrEP using established, well-characterized ARVs. To achieve this goal and to maximize efficacy, nanoparticle delivery systems require a careful design and characterization. In this manuscript we examined design criteria for four classes of nanomaterials, namely, polymeric nanoparticles, lipid-based nanoparticles, inorganic nanoparticles, and hybrid nanoparticles. Although inorganic nanoparticles can offer stability and relative ease of functionalization, they face challenges regarding biodegradation and biocompatibility, as well as capacity for payload depending on their chemical composition and structure. Lipid-based systems, like liposomes, are highly biocompatible and can carry both hydrophobic and hydrophilic drugs, but achieving sustained release can be difficult due to limitations in loading for some compounds and long-term stability. Polymeric nanocarriers offer excellent control over drug release properties, but it is challenging to control surface properties independently of the core properties and introduce targeting ligands. Consequently, each of these three distinct nanocarrier types has merits and disadvantages. The combination of different materials in one hybrid platform, the fourth type of nanomaterial covered in this manuscript, makes it possible to “mix and choose” the desirable properties of two or more materials to optimize the overall performance. For instance, the tunability of a drug-loaded polymer core can be combined with the biomimetic properties of a lipid membrane in lipid-coated polymer nanoparticles to generate a delivery platform with optimized core and surface properties. The hybrid approach and its ability to equip different polymer cores with specific surface properties may also help to alleviate one challenge for the clinical translation of ARV-loaded nanoparticles, that is, low drug loading. It is not uncommon for polymeric nanoparticles to show drug loading of below 10 wt %, which makes injection of high concentrations of nanoparticles necessary to achieve therapeutic efficacy [109]. The increased viscosity of very high nanoparticles concentrations can cause problems for intravenous injection and raises concerns about the possible nanoparticle-related side effects [109]. Maximizing the drug loading in an optimized polymer core remains a key parameter in the design of nanoparticle delivery platforms for reduced dose frequency in HIV PrEP.

## Data Availability

Data sharing is not applicable as no new data was generated or analyzed in this study.
